# Fabrication of Nano/Micro-Structured Electrospun Detection Card for the Detection of Pesticide Residues

**DOI:** 10.3390/foods10040889

**Published:** 2021-04-19

**Authors:** Kun Feng, Meng-Yu Zhai, Yun-Shan Wei, Min-Hua Zong, Hong Wu, Shuang-Yan Han

**Affiliations:** 1 Guangdong Province Key Laboratory for Green Processing of Natural Products and Product Safety, School of Food Science and Engineering, South China University of Technology, Guangzhou 510640, China; fengkun_89@163.com (K.F.); mengyu.zhai@outlook.com (M.-Y.Z.); 13650501669@163.com (Y.-S.W.); btmhzong@scut.edu.cn (M.-H.Z.); 2 College of Biosciences and Bioengineering, South China University of Technology, Guangzhou 510640, China

**Keywords:** nano/micro-structure, pesticide detection card, electrospinning, hydrophilic modification, sensitivity

## Abstract

A novel nano/micro-structured pesticide detection card was developed by combining electrospinning and hydrophilic modification, and its feasibility for detecting different pesticides was investigated. Here, the plain and hydrophilic-modified poly(ε-caprolactone) (PCL) fiber mats were used for the absorption of indolyl acetate and acetylcholinesterase (AChE), respectively. By pre-treating the fiber mat with ethanol, its surface wettability was improved, thus, promoting the hydrolysis of the PCL fiber mat. Furthermore, the absorption efficiency of AChE was improved by almost two times due to the increased hydrophilicity of the modified fiber mat. Noteworthily, this self-made detection card showed a 5-fold, 2-fold, and 1.5-fold reduction of the minimum detectable concentration for carbofuran, malathion, and trichlorfon, respectively, compared to the national standard values. Additionally, it also exhibited good stability when stored at 4 °C and room temperature. The food detection test showed that this nano/micro-based detection card had better detectability than the commercial detection card. Therefore, this study offers new insights into the design of pesticide detection cards, which also broadens the application of electrospinning technique.

## 1. Introduction

Pesticides are effective ingredients that have been extensively used to control agricultural diseases. However, their excessive use poses a challenge to the sustainable development of agriculture, and the administration of pesticide residues on the surface of food is harmful to human health. For this reason, considerable progress has been made recently in the determination of pesticides. Current approaches are mainly carried out by laboratory-scale analytical methods, such as HPLC, MC, GC, etc. [1,2]. Nevertheless, their inherent disadvantages, such as complicated pre-treatment, high costs, and time-consuming procedures, generally limit their applications [3]. Therefore, research into other effective, convenient, and reliable detection strategies has gained increasing attention in recent years [4,5,6]. Specifically, rapid detection based on enzyme acetylcholinesterase (AChE) inhibition has become widely accepted for pesticide residue analysis, ascribing to its simple principle and visual evaluation [7]. In this regard, the rapid detection card, generally made of glass fiber, qualitative filter paper, or absorbent paper, has been identified as the preferred and direct method for determining pesticides. Nevertheless, the challenge associated with these cards is the discrepancy between the sensitivity of the analysis and the increased awareness of food safety, making it imperative to seek new types of carriers for the production of detection cards.

Recently, extensive evidence has demonstrated the great potential of nano/micromaterials in the field of catalysis, owing to their unique structure and surface properties [8,9]. Herein, we try to further improve the conventional detection card’s performance by supplying the nano/micromaterials as the immobilization carrier for the enzyme and substrate. Electrospinning—a mild, convenient, versatile, and cost-effective technique for developing micro-/nano-structured vehicles—is associated with a broad range of applications in pharmacy, tissue engineering, food packaging, etc. [10]. Specifically, the superiority of electrospun fibers as the carrier for chemical biosensors and immobilization has been highlighted in recent years due to their desirable properties, such as a high surface area to volume ratio, porous structure, and tunable porosity [11,12]. However, electrospun fibers used as the detection card matrix for the pesticide detection card have been less explored, except for in our previous research study [13]. It is known that the electrospun fiber mat could not only be used as the encapsulation vehicle but also the absorption carrier [14]. Our published work has already designed a novel detection card by encapsulating the enzyme (AChE) and substrate (indolyl acetate, IA) into the electrospun nanofibers and we also demonstrated their proper role for determining pesticides more sensitively. Unfortunately, the deformation problem of this card, stemming from its hydrophilic feature, calls for the exploration of hydrophobic materials. Additionally, it is also interesting to determine the pesticide detection performance of the card by using the electrospun fiber mat as the absorption matrix for the enzyme and substrate.

Herein, a biocompatible and biodegradable polymer, Poly(ε-caprolactone) (PCL), was applied to prepare the detection card. To improve enzyme absorption on the fiber mat, hydrophilic modification of the hydrophobic PCL fiber mat was performed, and the related properties were investigated systematically. Subsequently, the plain and modified PCL fiber mat were employed to absorb IA and AChE, respectively. Furthermore, the key factors including the concentration of the enzyme and substrate, inhibition time, and color development time were optimized to improve the performance of the detection card. The detection ability of this card for different pesticides was examined and compared with the corresponding values specified by the Standardization Administration of China (SAC). Lastly, the feasibility of this card for detecting pesticide residues in real foods was studied by comparison with the commercial card. This study offers new access for the sensitive and convenient detection of pesticide residues, which is expected to inspire the further application of electrospinning in the field of food and agriculture.

## 2. Materials and Methods

### 2.1. Materials

Poly(ε-caprolactone) (PCL) was purchased from Sinopharm Chemical Reagent Co., Ltd. (Beijing, China). AChE (200 U/g, from head of fly) and IA were purchased from ShanghaiYuanye Bio-Technology Co., Ltd. (Shanghai, China). Trichlorfon and carbaryl were purchased from Aladdin (Shanghai, China). Malathion and carbofuran were purchased from the National Information Center for Certified Reference Materials (Beijing, China). The commercial rapid detection card was obtained from Dayuan Oasis Food Safety Technology Co., Ltd. (Guangzhou, China). Analytical pesticide standards (100 μg/mL) were stored at 4 °C and spiked to the desired concentrations. Samples of cabbage and apples were purchased from a local market (Guangzhou, China).

### 2.2. Preparation of the Detection Card Matrix

PCL solutions in the concentration range of 100–150 g/L were prepared by dissolving certain amounts of PCL into a mixed solvent of methanol and chloroform at different volume ratios (0, 1:9, 3:7, 5:5). Then, the sealed solution was placed on a magnetic stirrer and stirred at room temperature in the dark for more than one hour to obtain the stable solution. The conductivity and viscosity of different spinning solutions were measured by Brookfield digital viscometer (Model DV-II t Pro, Brookfield Engineering, Inc., Middleboro, MA, USA) and conductivity meter (DDS-11A, Shanghai Leici Chuangyi Instrument Co., Ltd., Jiading, Shanghai, China), respectively. Then, electrospinning was conducted by putting the solution into a plastic syringe (5 mL) with a 20-gauge steel needle which was connected to a high voltage power supply. The voltage and the distance between the needle tip and collector were set in the range of 11–17 kV and 11–15 cm, respectively. The injection rate was controlled by a syringe pump (NE-300, New Era Pump Systems Inc., Farmingdale, NY, USA) in the range of 1.5 to 3.5 mL/h. The fiber mat was fabricated at 24 ± 2 °C and under 55 ± 5% relative humidity for a period of time.

### 2.3. Hydrophilic Modification of the Matrix

Here, the PCL fiber mat was modified by one step or two steps of treatment. For the one-step modification, the PCL fiber mat was immersed in the NaOH solution with different concentrations (1 M, 2 M, 4 M, and 6 M) for a certain period of time, while the two-step modification was performed by initially infiltrating in the 70% ethanol for 15 min before the same NaOH immersion process. Then, the fiber mat was washed with deionized water and dried in a vacuum drying oven.

### 2.4. Characterization of the Matrix

The morphologies of the original and modified PCL fiber were observed by scanning electron microscopy (SEM, S-3700N, Hitachi High-Tech Ltd., Chiyoda-ku, Japan). Before the observation, the electrospun fiber mat was sputter-coated under vacuum conditions and observed at 15 kV. Then, the obtained SEM image was processed by the Nano Measure 1.2 software, and the distribution of the fibers was further calculated by analyzing around 50 fibers. The thickness of the fiber mat was measured by a digital thickness gauge (Syntek, Deqing Shengtai Electronic Technology Co., Ltd., Deqing, China).

The changes that occurred in the polymer molecules were examined by employing attenuated total reflection-Fourier transform infrared spectroscopy (ATR-FTIR) (VERTEX 70, Bruker Co., Ettlingen, Germany). All the spectra were recorded in the frequency region of 500–4000 cm^−1^ at a spectral resolution of 4 cm^−1^.

To characterize the surface wettability of the fiber mat, the surface contact angle tester (OCA40, DataPhysics Instruments GmbH, Filderstadt, Germany) was used to determine the contact angles of different samples. Briefly, the measurement was conducted by dropping 2 μL of deionized water onto the surface of the fiber mat with a drop rate of 2 μL/s. After 10 s, the contact angle of the fiber mat was measured by taking a screenshot. Each sample was tested 3 times, and the average value was calculated.

### 2.5. Optimization of the Immobilization and Determination Conditions

In this study, two fiber mats, namely the enzyme (AChE) fiber mat (AFM) and substrate (IA) fiber mat (IFM), were used for the preparation of the nano/micro-structured detection card. For optimizing the suitable AChE concentration for the preparation of AFM, the IFM was prepared by immersing the fiber mat in 5 mg/mL IA solution, and the cards were then dried in a vacuum oven. Similarly, the fiber mat was treated with 8 mg/mL of AChE solution when the optimal IA solution was investigated. Here, PBS (pH 7.4) and 1 mg/L of malathion were selected as control and positive sample, respectively. On the other hand, the absorption efficiencies of the fiber mat and other commonly used absorption materials (qualitative and quantitative filter paper) were examined by determining the amount of released enzyme from the carrier materials according to the Bradford method. Before doing this, the carrier materials were immersed into 5 mg/mL of AChE solution for 24 h and dried in a vacuum drying oven.

**Figure 1:**
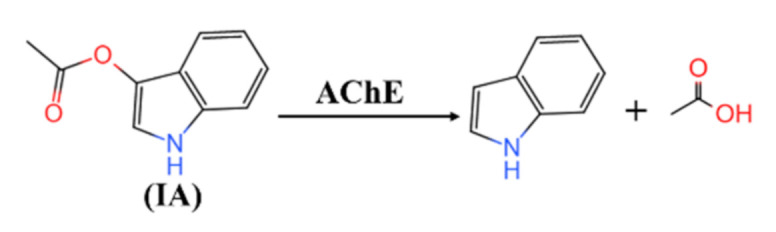
The reaction of indoxyl acetate hydrolysis, catalyzed by AChE.

The principle of this detection method is that AChE can catalyze the hydrolysis of colorless IA to produce indoxyl, as depicted in Figure 1, which then becomes blue due to the rapid oxidation by air. The blue color of indigo can be well distinguished with the naked eye. Hence, the existence of pesticides can be analyzed according to the color change as a consequence of the inhibition of the pesticide on AChE activity. On the basis of understanding the principle of this determination method, inhibition and color development time were two critical factors that significantly influenced the result. Herein, the inhibition time was optimized in the range of 5–15 min, where the color development time was set at 15 min. Similarly, the inhibition time was set at 10 min for exploring the appropriate color development time (5–25 min). PBS (pH 7.4) and 0.5 mg/L malathion were applied as control and positive samples, respectively.

### 2.6. Performance of the Detection Card and Its Real-Life Application

To evaluate the efficacy of this detection card, a series of concentrations of these two classical pesticides, including organophosphorus (OP) (omethoate and malathion) and carbamate (CM) (carbaryl and carbofuran), were determined and compared with their corresponding low limit of detection values reported in SAC. In brief, the pesticides were diluted to a series of concentrations by PBS. An aliquot (50 μL) of sample solution was dropped on the AFM for analysis, and PBS served as the control group. All the detection procedures were performed according to the conditions optimized in the above section.

The storage stabilities of the detection card under 4 °C and room temperature (RT) were evaluated for 60 days. Malathion and PBS were served as the positive and control group to examine its detection efficacy periodically.

To further verify the determination performance of this card, organic cabbage and carrots were tested as samples with different concentrations of malathion (0, 5, 10, 20 μg/mL). Different volumes of malathion were sprayed on sample surfaces based on the sample weight (1 mL/g) and stored at room temperature for 24 h. Subsequently, 5 g of the samples were immersed in 10 mL PBS and then shaken by hand. After the stabilization for 2 min, the supernatant was analyzed using self-made and commercial rapid detection cards.

### 2.7. Statistical Analysis

Statistical analysis was performed using one-way analysis of variance (ANOVA). A value of *p* ≤ 0.05 was considered statistically significant.

## 3. Results and Discussion

### 3.1. Preparation of the Nano/Micro-Structured Immobilization Matrix

**Figure 2:**
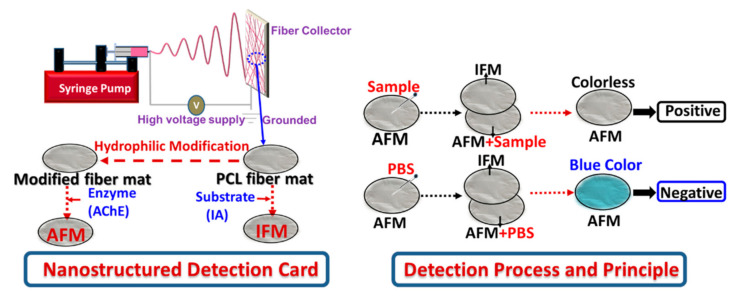
Preparation process and pesticide determination principle of the nano/micro-structured detection card.

Recently, the improved attention on food safety and pursuit of the sustainable development of the agricultural industry has promoted the exploration of efficient and convenient pesticide detection approaches. Specifically, our group has creatively investigated the potent application of electrospinning in designing rapid detection cards to determine different pesticides. The detection card was composed of two electrospun PVA fiber mats, in which the enzyme and substrate were encapsulated. Furthermore, it was found to be able to efficiently and sensitively detect different pesticides. However, the high hydrophilicity of PVA made the fiber mat easier to deform, which would impede its application to some extent. Given this situation, it is meaningful to investigate the performance of the detection card, taking a hydrophobic material as the card matrix. Additionally, electrospinning was also supposed as a proper absorption matrix in the field of analysis [15]. However, knowledge of its capability for use as a detection card is lacking. Therefore, as shown in Figure 2, this study offered another novel route for the preparation of pesticide detection card by electrospinning, where the electrospun fiber mats were employed as the immobilization matrix for the absorption of the enzyme (AChE) and substrate (AI) and the corresponding detection principle was depicted in Figure 2.

Here, an environmentally friendly material, PCL, was utilized to fabricate the detection card matrix, owing to its proper biodegradable and hydrophobic properties [16]. To successfully obtain the electrospun fiber mat, the appropriate solvent for PCL electrospinning should be initially investigated. It is known that solvents with low boiling points are more suitable for electrospinning [17]. Hence, a commonly used solvent, chloroform (CHCl_3_), was selected for hydrophobic PCL electrospinning, and methanol (CH_3_OH) was further blended with it to decrease the fast evaporation of the solvent during the electrospinning process because of the lower boiling point of the CHCl_3_ [18]. Furthermore, as shown in Table S1, the conductivity and viscosity of the solution were increased with the increase in the ratio of the CH_3_OH, which was similar to a previous study [19]. The fiber with a good morphology was obtained at the volume ratio of 3:7 (CH_3_OH: CHCl_3_) (Figure S1). Then, the suitable concentration of PCL for electrospinning was examined, and it was found that smooth fibers were obtained when the concentration was 125 g/L (Figure S2). The balance between the conductivity and viscosity, which was one key factor that would significantly influence the electrospinnability, was disrupted when the PCL concentration was 100 or 150 g/L, resulting in the beaded fiber morphology [20]. Thereby, 125 g/L of PCL concentration prepared in a blended solvent (CH_3_OH: CHCl_3_ = 3:7, *v*/*v*) was adopted for the preparation of the card matrix.

Additionally, the influence of different electrospinning parameters, including spinning voltage, distance, and injection rate, on the fiber morphology was studied. The SEM images of the fibers prepared under different parameters and their relevant fiber diameter distributions are shown in Figures S3–S5. It can be seen that smooth and uniform fibers were produced when the voltage was 13 kV. Lower voltages result in the accumulation of the electrospinning solution at the tip of the needle due to the weak electrical force [21]. While a high applied voltage increases the rate at which a polymer filament is drawn out of the Taylor cone, resulting in greater fiber elongation and a reduced fiber diameter [22,23]. In addition, fiber morphology was improved with the increase in the flow rate, but further increases in the flow rate could obtain thicker fibers. The reason behind this phenomenon was that the flow rate generally determined the amount of electrospinning solution at the tip of the needle and a higher flow rate would produce larger droplets and result in a lower charge density required for electrostatic repulsive forces to overcome the surface tension of the solution and the extra solution cannot be stretched during the electrospinning process [24]. Similarly, good fiber morphology was achieved when the distance was 13 cm. Higher or lower than this value would change the electric field distribution, disturbing the balance between the electrical force and surface tension [25]. Overall, the appropriate parameters for preparing of the PCL fiber mat were a spinning voltage of 13 kV, distance of 13 cm, and injection rate of 2.5 mL/h. The average diameter of the fibers fabricated under this condition was 1.07 μm.

### 3.2. Hydrophilic Modification of the Matrix and Its Characterization

**Figure 3:**
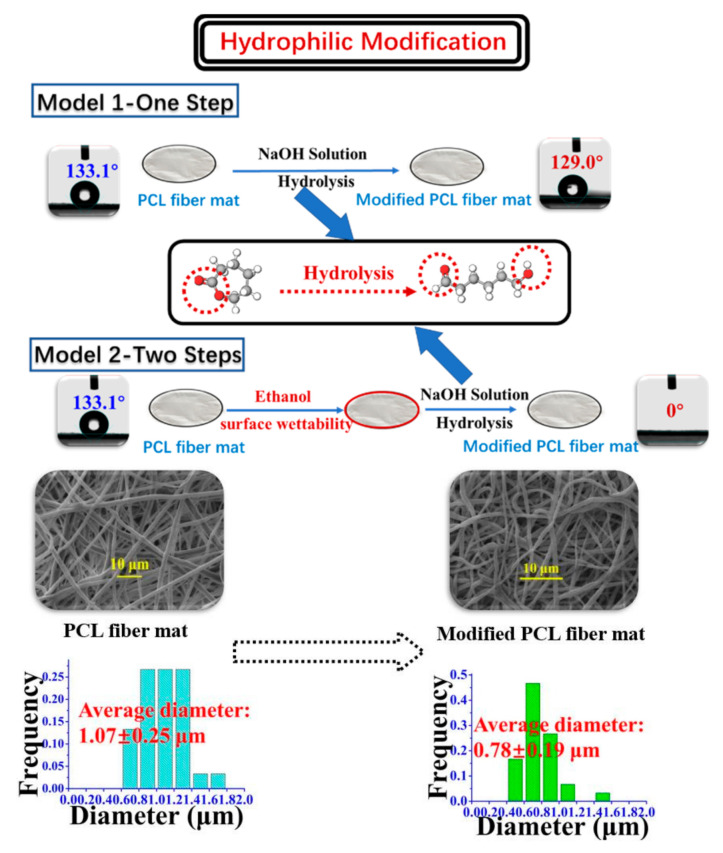
Two models used for the hydrophilic modification of PCL fiber mat and the properties of plain and modified fiber mat (*n* = 50).

In this study, the detection card consisted of an enzyme (AChE) loaded fiber mat (AFM) and a substrate (IA) loaded fiber mat (IFM). The AFM was fabricated through the absorption of the AChE onto the fiber mat. Unfortunately, the absorption efficacy was low because of the hydrophobic property of the electrospun PCL fiber mat. A previous study indicated that the absorption efficiency of the enzyme could be remarkably enhanced with an increase in hydrophilicity [26,27]. Therefore, hydrophilic modification of the PCL fiber mat might be a proper way to improve the detection performance of the designed detection card. To our knowledge, there were different approaches for the hydrophilic modification of PCL materials [28,29,30]. Nevertheless, alkaline hydrolysis was considered here on account of its simple and efficient features and two corresponding hydrophilic modification models, shown in Figure 3, were systemically investigated in this part. It was found that the contact angle changed from 133.1° to 129.0°, demonstrating that the hydrophilicity of the fiber mat was improved by direct treatment with the NaOH solution. This result could be attributed to the breaking of the easter linkages along the polymer backbone when the PCL fiber mat was immersed in the NaOH solution, leading to hydroxyl and carboxylic acid groups being exposed at the polymer surface [31,32]. Similar hydrophilicity modifications through the hydrolysis by alkaline have been conducted for other hydrophobic polymer membranes [33,34,35]. Noteworthily, as depicted in Model 2, the contact angle of the PCL fiber mat changed dramatically from 131.1° to 0° when it was presoaked with ethanol, which indicated that the surface of the fiber mat suffered a transformation from hydrophobicity to hydrophilicity. This phenomenon can be ascribed to the fact that the ethanol pretreatment can improve the surface wettability of the fiber mat, as described in a previous study [36]. If the NaOH solution was directly applied for the modification, the interaction between the NaOH solution and the PCL fiber would be limited because of its hydrophobic surface. However, the fiber mat can be pre-infiltrated when it was treated with ethanol, which was more conducive to the interaction between the PCL molecule and NaOH. Additionally, it is noteworthy that the size of the fiber also decreased from the microfiber to nanofiber scale after modification, while maintaining overall fiber structure. This structure change is favorable in the immobilization of the enzyme. Hence, Model 2 was utilized to modify the hydrophobic fiber mat. The influence of the NaOH concentration and the related hydrolysis time on the properties of the fiber mat were subsequently investigated.

**Figure 4:**
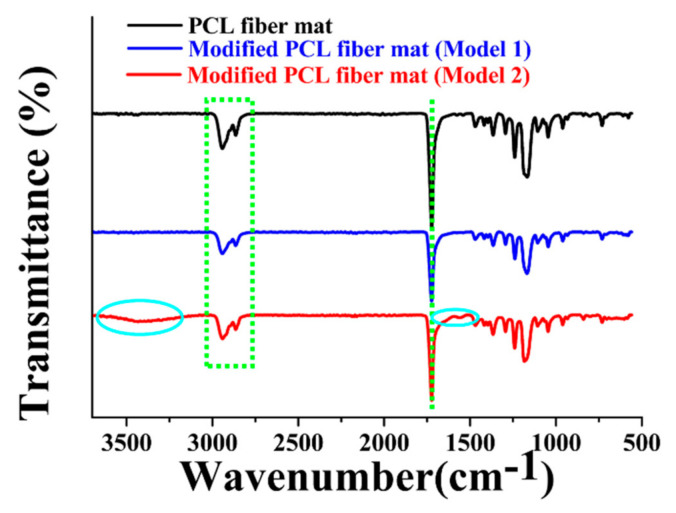
ATR–FTIR spectra of different samples.

To further illustrate the hydrolysis behaviors of the PCL fiber mat through two different modification models, ATR-FTIR was employed to characterize the interactions that occurred in the PCL molecule by observing the changes of the specific peaks. The spectra of different fiber mats are displayed in Figure 4. The intense peaks at 1723 cm^−1^ that appeared in different samples represented the presence of the ester carbonyl group (-CO stretching) in PCL polymer. Additionally, peaks at 2865 and 2942 cm^−1^ were related to the asymmetric and symmetric CH_2_ stretching. Apart from these characteristic peaks, there were two extra bands in the spectrum of modified fiber mat (model 2), one was at the wavenumber range of 1500–1700 cm^−1^ (C=O group) and the other was at the wavenumber range of 3250–3750 cm^−1^ (OH stretching vibrations). This can be explained that the presence of -OH (hydroxyl) functional group and -COOH (carboxyl) group in the PCL membrane after the hydrolysis of the ester carbonyl group [37]. Furthermore, it was concluded that Model 2 was more beneficial for the modification of the PCL fiber mat.

**Figure 5:**
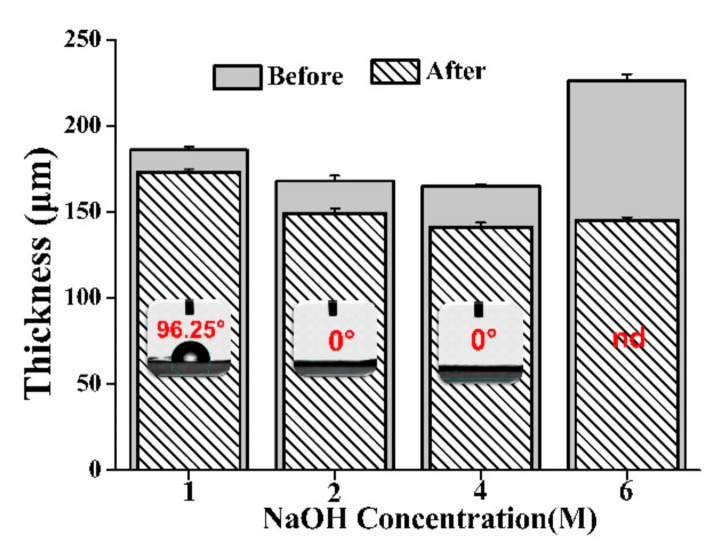
Effect of NaOH concentration on the thickness and contact angle of fiber mat before and after modification (nd, not determined, *n* = 3).

From the hydrolysis results displayed in Figure 5, it is clear that the thickness of the fiber mat decreased after treatment with NaOH, and the surface of the fiber mat could successfully be changed from hydrophobic to hydrophilic when the concentration of the NaOH solution was above 2 M. During the modification process, the fiber surface suffered different degrees of hydrolysis. The higher the NaOH concentration, the higher the degree of fiber hydrolysis. During the hydrolysis procession, the contact angle was decreased, probably due to the increase in surface free energy [38]. When the specific surface free energy of the fiber mat was increased to a critical value, the droplets dripping onto the surface of the fiber mat could easily penetrate into the fiber mat. However, further improving the concentration of the NaOH solution, the thickness of the fiber mat was seriously decreased and became fragile. Herein, 2 M and 4 M were determined as the suitable modification concentration to obtain the hydrophilic matrix.

**Figure 6:**
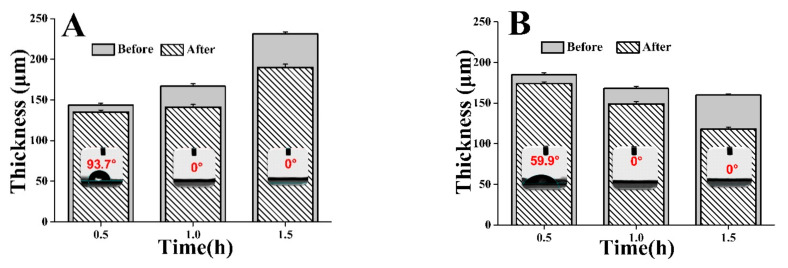
Effect of NaOH concentration and hydrolysis time on the thickness and contact angle of fiber mat before and after modification ((**A**), 2 M NaOH; (**B**), 4 M NaOH; *n* = 3).

Apart from the concentration, the effect of the treatment time on the properties of the modification fiber mat was examined. As displayed in Figure 6, 39.4° and 73.2° of reduction in the contact angle of the fiber mats occurred when the fiber mat was treated with 2 M and 4 M NaOH solution for 0.5 h, respectively, and the obtained fiber mats were still hydrophobic. Meanwhile, a considerable change in the wettability occurred when the hydrolysis time was longer than 0.5 h and the contact angles of these two modified fiber mats were both 0°, which led to an understanding that the improvement of the hydrophilicity of the modified fiber mat could be achieved by postponing the hydrolysis time. As the degree of fiber hydrolysis increased, the polar group hydroxyl and carboxyl content on the surface gradually increased. Additionally, many dips appeared on the fiber surface, which was in accordance with another similar study [39]. The increase in surface roughness may contribute to the hydrophilic property of the fiber mat [40]. Hence, appropriate modification was performed with the 2 M NaOH solution for 1 h.

### 3.3. Fabrication and Measurement Conditions of the Detection Card

**Figure 7:**
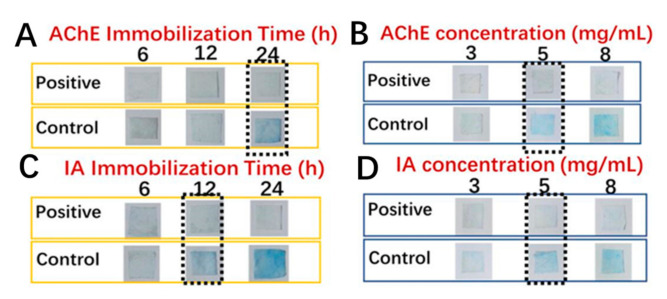
Color development of the detection card prepared under different conditions (**A**), AChE concentration 5 mg/mL, IA concentration 8 mg/mL, IA immobilization time 24 h; (**B**), AChE immobilization time 24 h, IA concentration 8 mg/mL, IA immobilization time 24 h; (**C**), AChE concentration 5 mg/mL, AChE immobilization time 24 h, IA concentration 8 mg/mL; AChE immobilization time 24 h; (**D**), AChE concentration 5 mg/mL, AChE immobilization time 24 h, IA immobilization time 12 h).

**Figure 8:**
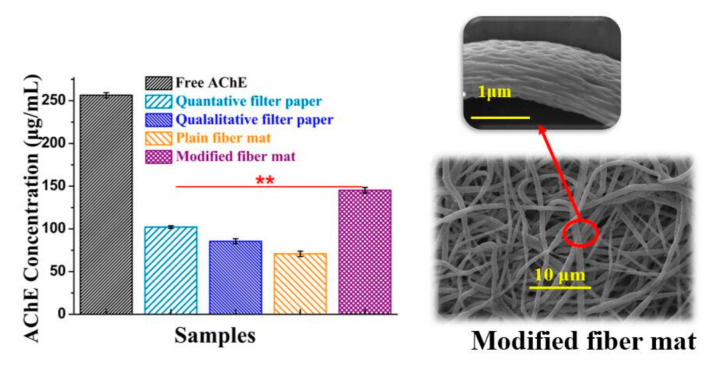
Absorption amounts of AChE for different matrixes and the surface morphology of modified fibers (*n* = 3, **, *p* < 0.01).

The rationale for this detection card is based on the hydrolysis of the substrate (IA) by the acetylcholinesterase (AChE) to produce indole phenol, which then becomes blue indigo due to the oxidization. In contrast, the card will be colorless if the AFM is treated with a positive sample (pesticide) as the enzyme activity can be inhibited by the pesticide. Here, to obtain a reliable detection approach, it is essential to investigate the influence of the two vital factors (absorption time and enzyme/substrate concentration) on the detection performance of this card and eventually to figure out the appropriate synthesis conditions. Here, the detection result could be obtained by simply distinguishing the color of the detection card with the naked eye. As shown in Figure 7, it appeared that the suitable immobilization time and concentration for the AChE were 24 h and 5 mg/mL, respectively. Less immobilization time or a lower concentration of AChE would result in a false-positive result. Likewise, 12 h and 5 mg/mL were found to be the proper time and concentration for the immobilization of IA to obtain a reliable detection result. On the other hand, considering the absorption efficiency of the AFM, the absorption amount of AChE for the commercial filter papers, plain and modified PCL fiber mats were determined and compared. As shown in Figure 8, the modified fiber mats showed a significantly higher absorption efficiency than the other two commercial applied filter papers (*n* = 3, *p* < 0.05), which may be attributable to the fact that the special nano-structure was more beneficial for the immobilization of the enzymes. More importantly, the fiber mat exhibited an improved absorption efficiency after the hydrophilicity modification, and a nearly 2-fold enhancement of the absorption amount was achieved. The probable reason was that the hydrophobicity property could hamper the interaction between the enzyme and the matrix. Moreover, the fiber morphology changing from microfiber to nanofiber after the modification might be another vital contributor to the improved loading efficiency of the enzyme. Furthermore, the pits on the surface of the modified fiber would naturally increase the specific area, thus, promoting the immobilization of the enzyme.

**Figure 9:**
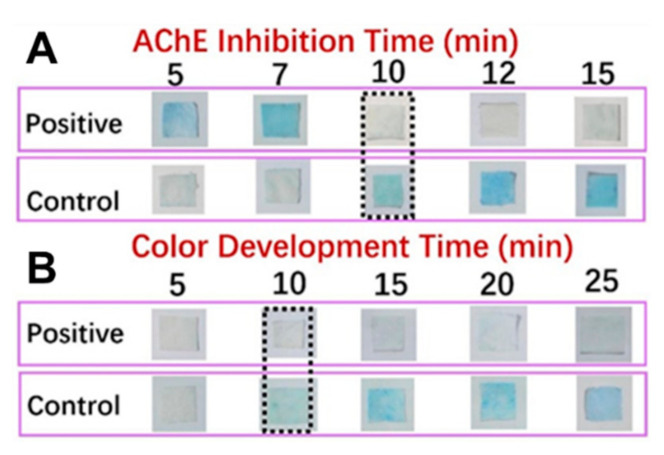
Color development of the detection card prepared under different conditions (**A**), color development time 20 min; (**B**), AChE inhibition time 10 min).

In addition to the card matrix preparation, the measurement parameters, mainly including enzyme inhibition time and color development time, are particularly important for the precise determination of the pesticides. It was known that the substrate IA could be decomposed into indole phenol by the catalysis of AChE, and then blue indigo would be formed after oxidization. In contrast, the inhibition of AChE activity by the pesticide would result in the colorless appearance of the detection card. The inhibition time, referred to as the reaction time between the sample and AFM and the color development time, indicated the time that AFM with IFM. Generally, the inhibition time has a considerable influence on color development. If the inhibition time was too short, the enzyme could not react with the sample sufficiently, leading to a negative result. Nevertheless, if the inhibition time was too long, it would be time-consuming and decrease productivity. On the other hand, time was required for AFM to react with IFM in order to develop the distinct blue color. Similarly, a shorter or longer color development time would limit the real-life application of the detection card. For this reason, the exploration work of these two factors was conducted, and the related results were displayed in Figure 9. The suiFireaction condition was found to be an inhibition time 10 min and a color development time 10 min, which was superior to the commercial detection card.

### 3.4. Detection Performance of the Detection Card and Its Real-Life Application

Nowadays, consumers would like to select foods with fewer pesticides. Pesticide detection with high sensitivity seems to be the preferred strategy and would have great application potential in the food and agricultural industry. To check the detectability of this card, four pesticides representing two main types of pesticide, organophosphorus and carbamate, were selected to be tested. Additionally, the minimum detectable concentration of this card for each pesticide was compared with its corresponding limit of detection (LOD) value specified by SAC [41]. Results depicted in Table 1 elucidated that this nano/micro-structured detection card exhibited a comparative value with the corresponding LOD values. It is noteworthy that 5-fold, 2-fold, and 1.5-fold reductions in the detectable concentrations of the rapid detection card for carbofuran, malathion, and trichlorfon were obtained compared to the national standard value, suggesting that this developed card could be able to detect these types of pesticides.

**Figure 10:**
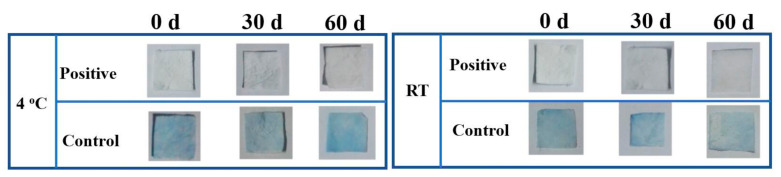
Storage stability of the detection card under 4 °C and room temperature (RT).

The shelf life of the as-prepared detection card is another important factor that influences its real-life applications. In this study, the storage stability of this detection card under 4 °C and RT were investigated. As shown in Figure 10, all the detection cards in PBS groups had a blue color, while other detection cards were colorless, demonstrating that this detection card had good stability. It could effectively detect the pesticide even though they were stored for 60 days. Hence, this desirable property of the self-made card also contributes to its real-life application.

To verify the real-life performance and practicability of this self-made detection card, pesticide residues on cabbage and carrot were measured according to the above-mentioned conditions, and comparisons were also performed with the commercial detection card. As shown in Table 2, the commercial card cannot detect the malathion even at its concentration of 20 µg/mL, since the control group still showed a blue color. However, the obtained detection card could accurately detect 5 µg/mL malathion, indicating the existence of pesticide on the cabbage. Similarly, the detection results for the carrot also revealed that this self-made detection card had a better detectability than the commercial card. The card with a nano/micro-structure could determine pesticides on the real vegetables accurately and efficiently. Therefore, this study opens up a new way to develop the pesticide rapid detection vehicle, promoting the sustainable development of the agricultural and food industry.

## 4. Conclusions

To satisfy the improved requirement of safety food, a novel nano/micro-structured pesticide detection card was creatively fabricated by taking the electrospun fiber mat as the card matrix. This detection card has good storage stability and a low minimum detectable concentration, the application of which involves a short reaction time, simple operation, and minimum use of human and material resources as compared to the traditional detection approaches. In addition, experiments with real samples also demonstrated its feasibility and superiority on pesticide detection over the commercial card. More importantly, this detection method can even be performed by nonprofessional individuals, making it more in line with the market requirement. Therefore, the present study offers a new route for designing a rapid detection card for pesticides, which could promote the application of the electrospinning technique and nano/micro material in the agricultural and food industry.

## Figures and Tables

**Table 1:** The minimum detectable concentrations (MDC) of the card for different pesticides and the corresponding limit of detection (LOD) values specified by Standardization Administration of China (SAC).

Pesticide		LOD (mg/L)	MDC (mg/L)	Color Development			
Organophosphorus	Trichlorfon	0.3	0.2	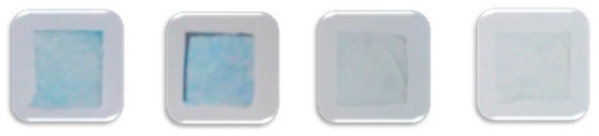			
				0	0.1	0.2	0.3
	Malathion	2.0	1.0	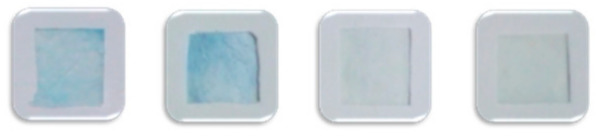			
				0	0.5	1.0	2.0
Carbamate	Carbaryl	2.5	2.5	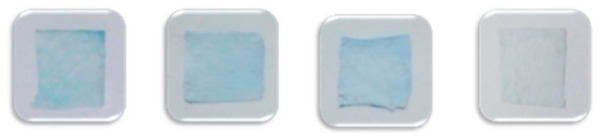			
				0	1.0	2.0	2.5
	Carbofura	0.5	0.1	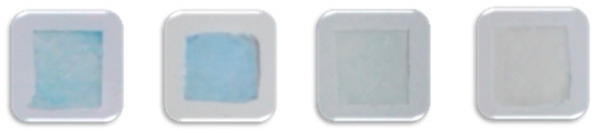			
				0	0.05	0.1	0.5

**Table 2:** Color development results for real-life food detection.

Sample	Detection Card	Spraying Concentration of Malathion (μg/mL)			
		0	5	10	20
Cabbage	Commercial	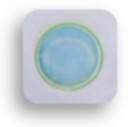	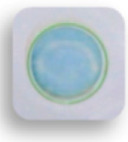	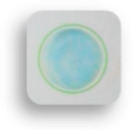	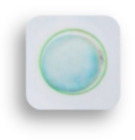
	NMF	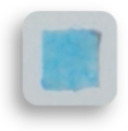	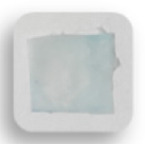	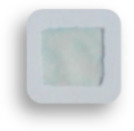	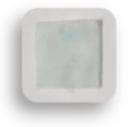
Carrot	Commercial	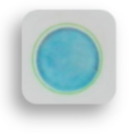	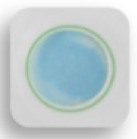	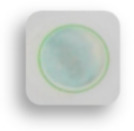	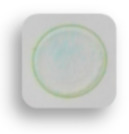
	NMF	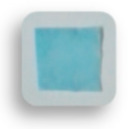	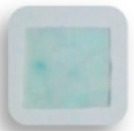	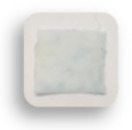	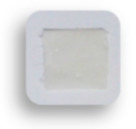

Note: NMF, nano/micro-structured detection card.

## Data Availability

Data are contained within the article or supplementary material.

## Supplementary Materials

The following are available online at https://www.mdpi.com/article/10.3390/foods10040889/s1, Figure S1: SEM images of electrospun fibers at different ratios of CH_3_OH to CHCl_3_ (the electrospinning conditions were: voltage of 13 kV, flow rate of 2.5 mL/h and distance of 13 cm); Figure S2: SEM images of electrospun fibers at different concentrations of PCL (the electrospinning conditions were: voltage of 13 kV, flow rate of 2.5 mL/h and distance of 13 cm); Figure S3: SEM images and fiber diameter distributions of PCL fibers under different electrospinning voltages (the electrospinning conditions were: flow rate of 2.5 mL/h and distance of 13cm); Figure S4: SEM images and fiber diameter distributions of electrospun PCL fibers under different flow rates (the electrospinning conditions were voltage 13 kV and distance 13 cm); Figure S5: SEM images and fiber diameter distributions of electrospun PCL fibers under different distances (the electrospinning conditions were: voltage of 13 kV and flow rate of 2.5 mL/h); Table S1: Effect of PCL concentration and volume ratio of CHCl_3_ to CH_3_OH on the properties of electrospinning solution.
