# Supplementary material for: Fabrication of Nano/Micro-Structured Electrospun Detection Card for the Detection of Pesticide Residues
Source: Foods. 2021 Apr 19;10(4):889. doi: 10.3390/foods10040889 (PMC8073816; doi:10.3390/foods10040889)
Supplement: Supplementary file 1 [file foods-10-00889-s001.zip › foods-1167491-supplementary/Manuscript/Supplementary Material.pdf]

Supplement Information

Fabrication of nano/micro-structured electrospun detection card for the detection of pesticide residues

Kun Feng [a], Meng-Yu Zhai[a], Yun-Shan Wei[a], Min-Hua Zong[a], Hong Wu[a*], Shuang-Yan Han[b*]

[a] School of Food Science and Engineering, Guangdong Province Key Laboratory for Green Processing of Natural Products and Product Safety, South China University of Technology Guangzhou 510640, China

[b] College of Biosciences and Bioengineering, South China University of Technology Guangzhou 510640, China

*Corresponding authors. E-mail addresses: bbhwu@scut.edu.cn (H. Wu), syhan@scut.edu.cn (S.-Y. Han).

Table S1 Effect of PCL concentration and volume ratio of $CHCl_3$ to CH_3OH on the properties of electrospinning solution (n=3).

PCL (g/L)	$CH_3OH:CHCl_3$	Viscosity (mPa·s)	Conductivity (μs/cm)
100	3:7	23.1±0.2	1.163±0.006
125	3:7	45.4±0.3	1.302±0.014
150	3:7	84.5±0.2	1.417±0.007
125	0	28.2±0.4	0.002±0
125	1:9	35.9±0.4	0.178±0.004
125	3:7	45.4±0.1	1.417±0.011
125	5:5	59.9±0.3	1.734±0.007

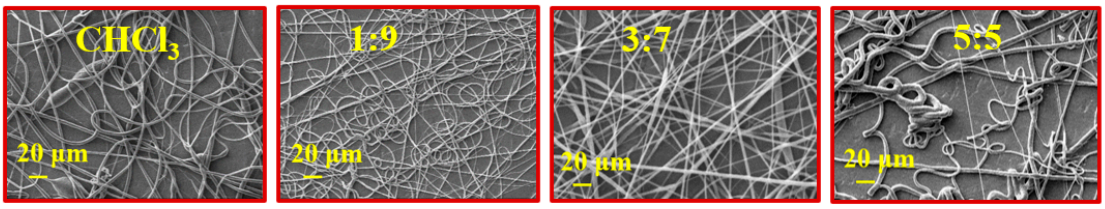

Figure S1. SEM images of electrospun fibers at different ratios of CH_3OH to $CHCl_3$ (the electrospinning conditions were: voltage of 13 kV, flow rate of 2.5 mL/h and distance of 13 cm).

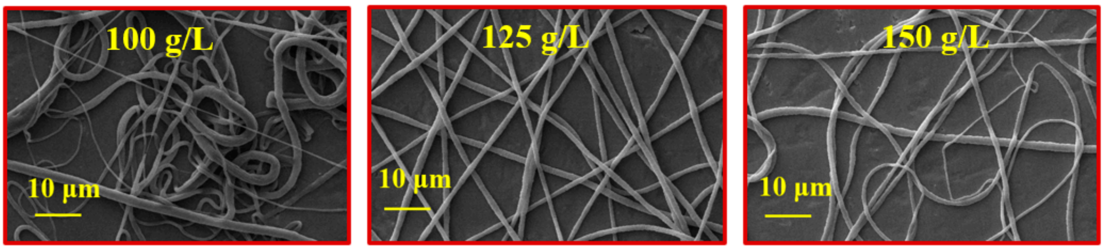

Figure S2. SEM images of electrospun fibers at different concentrations of PCL (the electrospinning conditions were: voltage of 13 kV, flow rate of 2.5 mL/h and distance of 13 cm).

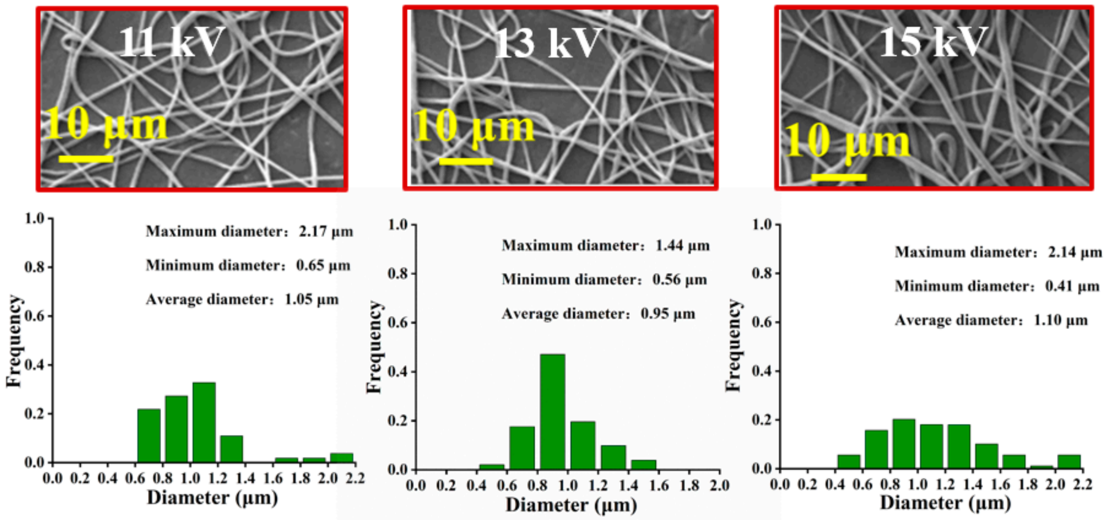

Figure S3. SEM images and fiber diameter distributions of PCL fibers under different electrospinning voltages (the electrospinning conditions were: flow rate of 2.5 mL/h and distance of 13cm).

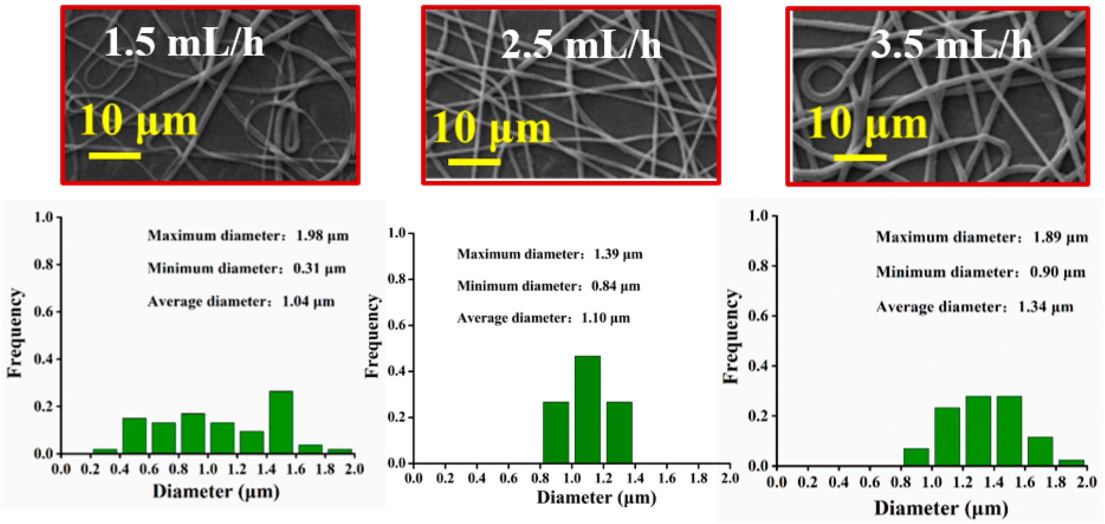

Figure S4. SEM images and fiber diameter distributions of electrospun PCL fibers under different flow rates (the electrospinning conditions were: voltage of 13 kV and distance of 13 cm).

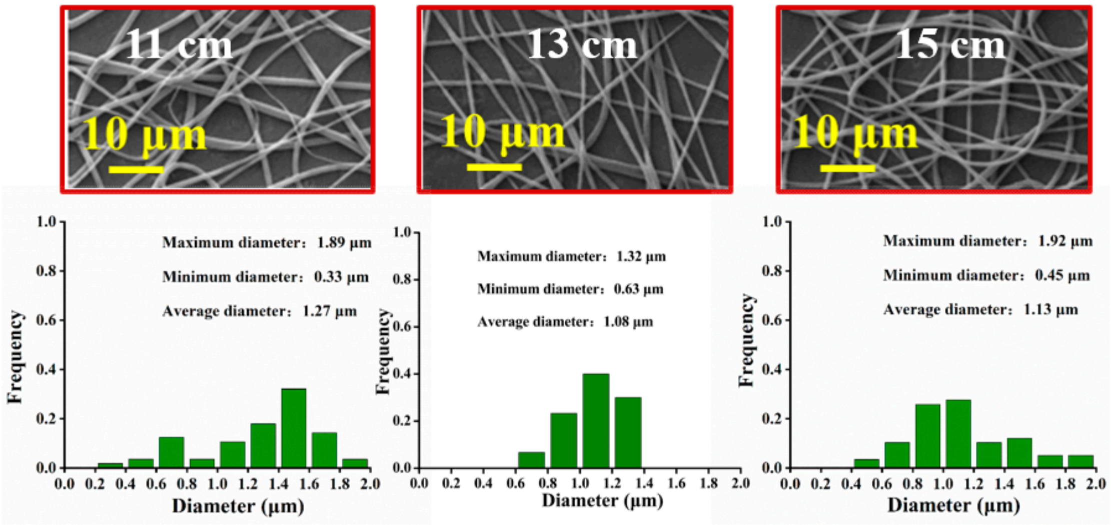

Figure S5. SEM images and fiber diameter distributions of electrospun PCL fibers under different distances (the electrospinning conditions were: voltage of 13 kV and flow rate of 2.5 mL/h).
